# Introducing occupational health management in the German Armed Forces

**DOI:** 10.1093/heapro/dax035

**Published:** 2017-07-03

**Authors:** Ute Latza, Eva Hampel, Markus Wiencke, Michaela Prigge, Andreas Schlattmann, Sabine Sommer

**Affiliations:** 1 Division of Work and Health, Federal Institute for Occupational Safety and Health (BAuA), Noeldnerstr. 40-42, Berlin, Germany; 2 WiDi-Kontor, Scientific and Research Services, Seewartenstraße 10, Hamburg, Germany; 3 Division of Policy Issues and Programmes, Federal Institute for Occupational Safety and Health (BAuA), Noeldnerstr. 40-42, Berlin, Germany; 4 Department for Sport Sciences, Sport Management, Bundeswehr University Munich, Werner-Heisenberg-Weg 39, Neubiberg, Germany

**Keywords:** process evaluation, qualitative methods, worksite, healthy settings, implementation

## Abstract

Holistic approaches to workplace health promotion (WHP) within the military setting are challenging. In 2015, the German Ministry of Defense initiated a 6-month pilot study of WHP in the Federal Armed Forces. The pilot study was to identify organizational challenges that should be addressed before the Ministry implemented a comprehensive occupational health management policy in all departments. Eleven diverse departments were selected to participate in a WHP program that addressed physical activity, diet, stress management and addiction prevention. As part of the evaluation concept, we interviewed coordinators, and department heads focusing on transfer factors from the perspective of the implementers. All coordinators and their department heads or deputies participated in semi-structured face-to-face on-site interviews. The data were analyzed based on qualitative content analysis. The coordinators (officers with sports science degree) seemed fully prepared and capable to master the new task. They experienced difficulties in adapting WHP activities to local structures and needs, and complications in administering modular activities. Department heads described conflict regarding human resources between the military mission and the implementation of WHP. Commitment to WHP was a strong facilitator. The interviews identified various barriers related to support by middle management (supervisors) and specific work conditions (e.g. shift work). If occupational health management is to be successfully implemented on a large scale, conceptional and practically collaboration is necessary between WHP and occupational safety and health, and organization and leadership, respectively. Supervisors will benefit from open communication about compensation for the release time of their subordinates to attend WHP.

## BACKGROUND

Workplaces are promising settings to influence health behaviors and to link workplace health promotion (WHP) activities with other activities in the workplace to support worker health and wellbeing, such as occupational safety and health (OSH). As yet, there is a lack of scientific literature from diverse settings that introduce and evaluate key concepts of health promotion (WHO, 1986) in culturally diverse settings (Van den Broucke, 2016).

In 2015, the German Ministry of Defense initiated a pilot study of a WHP to provide information about organizational challenges that need to be addressed before the Ministry implemented a comprehensive occupational health management (OHM) policy. Based on the WHO definition of health (WHO, 1986) and the concepts of salutogenesis, the overall objective of the policy is to provide working conditions under the responsibility of the Ministry that promote health and wellbeing to fulfill the mission of the Federal Armed Forces. The whole system approach is integrated into the organizational structure with the three pillars WHP, OSH, and organization and leadership (Bundeswehr, 2013). Similar to the US ‘Army Health Promotion’ program (AR 600-63, 2015), OHM is embedded into a larger political agenda (Bundesministerium der Verteidigung, 2014). The purpose of the ‘Agenda Attractiveness’ is to attract qualified, motivated, and resilient soldiers and civilians. The economic dimension of supportive environments for health (Sundsvall Conference, 1991) was covered by explitely dedicating diverse resources to develop the Federal Armed Forces as a healthy workplace. This included re-allocation of positions for WHP coordinators. Further, all employees in piloting departments were enabled to participate in WHP activities during two hours of working time per week. In this article, we present specific results showing challenges they faced implementing the WHP pilot study. 

The German Armed Forces are the third largest in the European Union, after Great Britain and France, with a workforce of about 250 000 employees, two-thirds of which are in the military services. The military organization is characterized by a strong hierarchy and clear command structures. The complex infrastructure includes all military branches, civil service units, and facilities and staff for sport, health, psychological and social services. 

Eleven diverse departments were selected to participate in the pilot study. They represented the diversity within the portfolio of the armed forces and civil units under the responsibility of the Ministry. During the 6 months of pilot testing, sport scientists from the Federal Armed Forces were enabled to coordinate the WHP implementation in the four priority focus areas (physical activity, diet, stress management/sleep quality and addiction prevention).

A scientific steering group accompanied the WHP pilot study and the different qualitative and quantitative evaluations (repeated employee surveys for comprehensive needs and risk assessment with effect evaluation, partial evaluations of the priority focus areas such as diet, process evaluation with stakeholder interviews and regular monitoring) (Franke, 2015; Sammito *et al.*, 2015). As part of the evaluation concept, the aim of the presented process evaluation was to identify transfer factors from the perspective of the implementers. For this purpose, we interviewed the coordinators who implemented the WHP and their department heads. We were especially interested in interviewees’ thoughts about general and context-specific facilitating and hindering factors they observed in conducting the pilot. By presenting the results in a framework of systematically compiled transfer factors (Kliche and Touil, 2011), we aimed to identify the potential and limitations to the implementation of a comprehensive OHM within a complex hierarchical organization.

## METHODS

We invited all coordinators and their department heads to participate in semi-structured interviews. Between 5 May 2015 and 25 June 2015, we conducted face-to-face interviews on-site with the 11 coordinators and their 10 department heads or deputies. The departments represented all federal defense forces (Army, Navy, Air Force, Joint Support Services, and one military hospital within the Joint Medical Service), governance (the Federal Ministry of Defence), and civil administrative units.

We used different interview guides for the coordinators and the department heads. The questions were related to organizational aspects (e.g. WHP infrastructure and organizational culture), and WHP program aspects (e.g. enablement of coordinators to empower their target population, adaption of WHP activities to different contexts and participant needs, and marketing). We also tried to elicit suggestions for improvements when the program is applied on a large scale. The interviews were conducted individually, audio-recorded with interviewee permission and lasted ∼45–90 min.

Relevant parts of the interviews were transcribed or directly paraphrased and coded using an application for qualitative data analysis (MAXQDA version 11, VERBI GmbH, Berlin, Germany). We analyzed the coded and categorized text fragments with the content analysis method by Mayring (cf. Mayring, 2015). In addition to the queried topics, we identified novel themes in the coordinator interviews (e.g. the role of middle management) and department head interviews (e.g. shift-work as a special challenge). The transcriptions were cross-checked with the recordings to ensure accuracy and anonymity.

We used the framework of systematically compiled transfer factors by Kliche and Touil (Kliche and Touil, 2011) to critically reflect and generalize our results. The framework describes 38 factors related to barriers and facilitators for implementing activities of prevention and health promotion based on a systematic literature review (review of reviews).

## RESULTS

As the organizers intended, the departments differed considerably in duties (military or civil, administration/governmental, combat troops or supporting units), working conditions, size, competencies, age of the workforce and locations (rural or urban) (Sammito *et al.*, 2016). Nevertheless, all departments indicated both context-specific and similar facilitators and barriers.

### WHP program characteristics

#### Coordinators’ role and training

Before the program began in January 2015, the recruited coordinators (officers with sports science degree) were offered professional training and development. During a 5-week course in the German Armed Forces Sports School, they were enabled to coordinate the implementation of the WHP activities.

The interviews revealed that professional knowledge and personal skills were essential for coordinators. As sports scientists, they had knowledge about the biopsychosocial model of disease causation. As officers they knew the military culture. To successfully implement the program where they were assigned, they had to be familiar with the specific departmental hierarchies. Most coordinators reported that their military habit was vital: ‘A civilian would not be accepted. A reserve officer might work, but the combat troops would not accept someone who had no military background. You have to know how people tick here’.

The heads of the purely military departments agreed that coordinators should be active soldiers. In other departments, particularly civil departments, civilian employees or reserve officers from other academic disciplines (e.g. psychology) were considered as suitable future coordinators. Coordinators’ personal motivation, social skills and ability to improvise were considered essential. Department heads named creativity, flexibility, assertiveness, ability to motivate, empathy, persuasiveness, confidentiality and friendliness as relevant social skills: ‘You need someone who has an innovative and fun spirit, someone who likes people, feels comfortable in a crowd, likes to see things move forward, who doesn't quit or take the easy route, someone who can lead – but with a smile’.

The interviews provided convincing arguments regarding inhibiting factors especially on the middle management level (see ‘Leadership’ below). Coordinators were captains or lieutenants, so they often found it difficult to directly contact higher-ranking persons concerning WHP organization. During the training course, coordinators established a stable colleague network. They also met some of the department heads who attended a 2-day session during the training course at the German Armed Forces Sports School. Both sides described a positive and efficient direct cooperation between coordinators and department heads or deputies.

#### WHP program organization and processes

Only three coordinators had previous experience in their assigned departments; all others had to newly orientate themselves. As militaries, the coordinators clearly understood the culture and processes in military structures but experienced some difficulties in the Ministry. They could adapt their previous experiences to meet the new demands. The mainly civilian departments lacked enough trainers to guide the physical activities. Consequently, all coordinators but one had to serve as physical trainers, at least initially, which led to a high work load. A priority was thus to find new trainers within the organization and motivate them to develop their own training programs.

All officers are licensed physical trainers, but were often less available than expected. Their training skills often did not meet participants’ preferences, such as for popular yoga or fascia training that do not conform to German Armed Forces regulations. In the future, success will depend on having enough qualified and motivated physical trainers.

The interviews revealed that all departments will need to establish permanent contacts and a qualified local workplace health committee to select WHP activities appropriate to the different contexts (such as infrastructure) and needs of their target population (related to age, gender, occupation, rank, physical activity level and mental stress level). They would also benefit by having an information technology (IT) infrastructure that would allow coordinators to contact all employees personally. However, this was not always desired or even technically possible due to the IT infrastructure or security requirements. Although the department heads were advocates for WHP at kick-off events or in regular meetings with their management, more professional marketing support is needed in the future (e.g. establish a logo, advertise the program, and provide knowledge-management and information through an IT-platform).

The coordinators agreed that the impact of WHP activities should be evaluated scientifically.

#### WHP program activities

All departments implemented WHP activities, but sometimes the activities were underutilized. The coordinators used their freedom to adapt the WHP activities, particularly the physical activities, to meet the needs of the local population. One coordinator explained: ‘I went out with the soldiers to the training ground, took part in shooting practice,… . and observed typical grenadier activities. And from there I selected activities to incorporate health aspects to meet with their physical demands and help to prevent overload’. Thus, one coordinator concluded: ‘In the physical activity area, we not only reached the goal; we went far beyond it’.

The stress and addiction prevention activities had a standardized modular form that made implementation more difficult. In the future, more flexibility and freedom to adapt will be essential. Interviews revealed that some behavior-oriented nutrition activities could not be implemented as planned. Interviewees reported limited employee responses regarding addiction prevention activities, probably because of stigmatization fears.

### Organizational characteristics

#### Leadership

Decisions to introduce OHM and the WHP pilot began at the Ministry level, without involvement of the departments (top down). After they had given their consent to be part of the pilot phase, department heads were responsible for implementing the WHP program. During a 2-day training program at the German Armed Forces Sports School, they were enabled to advocate, enable and mediate OHM/WHP in their departments. This included discussions on their departmental resources, and WHP implementation schedules. Ten departments implemented the possible allowance of two hours WHP activities during working hours per week for all employees; one department allocated 1 h/week.

The armed forces and their civil units are organized according to distinctive hierarchies, clear command structures and authoritarian leadership. However, within the military culture leadership also comprises motivation, commitment, comradeship, employee-orientation and trust. ‘We are a very hierarchical organization… but we take our time to talk to the people because otherwise we cannot lead them’. The Ministry and all military units are under constant and fundamental reorganization, so the department heads described OHM/WHP implementation as a double-edged sword. Although they should and wanted to implement it, they wanted to show that they need more resources to do so. They described this core conflict also for middle managers.

All department heads regarded fulfilling the structural mandate as their first priority, as one department head stressed: *‘*Being part of the pilot study is important. Occupational health management is good for us, but we must meet our military mission. We are not doing sports for ourselves alone; this unit has to support the combat troops. I cannot resolve this decisional conflict for any officer’.

We did not ask explicitly about middle management roles in WHP implementation, but all coordinators and department heads mentioned middle management problems in various contexts. Employees had to secure supervisors’ permission to participate in WHP activities. Thus participation depended on employee motivations and supervisor decisions. Athletic supervisors tended to participate and support staff participation. However, supervisors were under pressure to show output- and number-oriented success. Middle managers were challenged to organize work to allow subordinate participation and to participate as examples, although each participating subordinate lost up to two working hours per week. Thus, middle managers had less personal working time and more organizing responsibilities. The situation was often exacerbated when supervisors had to compensate for the missing work capacities of physical trainers released from regular duty to prepare and conduct exercises. Thus supervisors generally saw WHP as extra work that could impede the fulfillment of core tasks. Consequently, middle management may have seen low relative advantages in participating or motivating their subordinates to participate. Although supervisors did not dissent overtly, our interviewees hinted that some supervisors informally undermined the project, such as by withholding information about WHP activities to their staff or making disdainful comments about WHP or persons participating in the activities. The department heads were aware that orders were conflicting with WHP: ‘Recently I gave new orders to the commander of deployment preparation and he said: “Well, seems like I can cancel my WHP participation to fulfill these orders.” The work could not be delayed, so he had to repeatedly cancel his participation’.

Department heads generally saw OHM as a change process requiring more investments in human resources. They generally favored OHM, but did not feel that it was sufficient to make the army a more attractive employer. Challenges related to the military culture were described: ‘Well, “health” and “psychosocial” are difficult topics in the federal defence forces because we are tough guys’. However, some department heads noticed changes in individual health and in the organizational culture *‘*…if I do OHM properly, it changes the departmental culture’.

#### WHP integration with occupational safety and health

In general, interviewees lacked knowledge of the role of OSH in OHM and did not understand why OSH experts participated in the (departmental) committee for health. Because of its normative nature, OSH may be inadequately adapted to employee needs. The department heads mentioned that while individual aspects of OHM already existed, the WHP activities would give the whole concept a ‘new drive’, especially the preventive approach. A department head saw a fundamental change in perspective: ‘Every soldier has experience with occupational safety like ear protection or safety shoes and knows that a psychologist can help with mental problems. So I was familiar with the individual aspects, but the OHM preventive approach was new to me’.

#### Infrastructure

In our analysis, we examined how the activities might be adapted to fit local working conditions. One department head summarized that ‘After six months it was obvious to me that no standard solution exists for all departments. One has to work with the framework and the core idea and use the given resources for establishing the best solutions for the soldiers and the civilian employees’.

In general, the coordinators could adapt the activities to the structures and needs in their respective departments in terms of time, space, frequency and duration. With the help of the department heads, the coordinators could arrange appropriate procedures. For example, when WHP activities took place outside department premises or working hours, specific permissions and instructions were required to ensure insurance protection. Some departments had to consider their exceptionally diverse jobs, working conditions and surroundings (e.g. on vessels in the harbor, special security zones and shift work) which meant that these employees could not be reached at all or only to a very limited degree. Particularly, employees in hospital shift work or in top security zones could not participate in consecutive or modular activities such as the provided courses in stress management or sleep coaching. Likewise, employees in shift work were not suitable as physical trainers for coordination reasons. Coordinators had to develop alternatives to meet time requirements, such as offering activities before and after shift work. The department heads had to develop procedures for ensuring that the time was recorded as working time.

The Federal German Armed Forces are characterized by their ability to respond rapidly to change. OHM is only one element in an ongoing fundamental process of organizational reorganization, as one department head explains: ‘We are in a permanent state of change. OHM is but one of many changes’.

## DISCUSSION

Equity in health requires the implementation of key concepts of health promotion (WHO, 1986) in culturally diverse populations (Van den Broucke, 2016). With the ‘Agenda Attractiveness’, the German Ministry of Defence addresses social, political and economic dimensions of supportive environments for health and wellbeing (Sundsvall Conference, 1991) within the topics leadership and work–family balance (e.g. leadership functions for men and women working part time, shared management positions, child care and teleworking) (Bundesministerium der Verteidigung, 2014) that may influence a cultural change. Healthy workplace is one of the topics on the agenda with a commitment to advocate health and wellbeing to fulfill the mission of the Federal Armed Forces. For this purpose, the Federal Armed Forces pilot-tested a WHP program as a precursor for later implementation of a comprehensive OHM that includes WHP, OSH and organizational development.

Valid evaluation is important for comprehensive government-funded health promotion (Brug *et al.*, 2011). Researchers rarely evaluate using mixed methods that generate both local and generalizable knowledge (Latza, 2011). As part of a larger evaluation concept (Franke, 2015; Sammito *et al.*, 2015), we interviewed coordinators and their department heads to gather their perceptions regarding facilitators and barriers in implementing the program. Our study provides local knowledge about organizational aspects and special needs for allocating resources in future large-scale implementation of the OHM. By presenting the influencing factors in a systematically compiled framework (Kliche and Touil, 2011), we further generate understandings about the potential and limitations for transferring the results to other organizations. The framework is comparable to the developed models in two subsequent systematic reviews (Wierenga *et al.*, 2013; Rojatz *et al.*, 2016) in which the facilitators and barriers are additionally assigned to different levels (e.g. levels of organization and context). Rojatz *et al.* (2016) further differentiate between intervention phases.

Interviewees talked about conflicts between OSH and the military mandate that emerged from a fundamentally different understanding of health risks. Large-scale implementation will require a conceptional and practical collaboration between WHP and OSH (safety experts, occupational health practitioners, disability managers), and organization and leadership, respectively. Although coordinators had the limited task of introducing WHP, organization and leadership, and OSH were already influenced by the pilot phase.

Coordinators must be enabled to adapt the WHP activities to the needs of their target populations and the local contexts. In the pilot phase, they could dynamically develop the physical activity exercises to fit the specific workplaces and the individual fitness of employees. In the future this flexibility will also be necessary in the other WHP areas (e.g. stress management). Considering that the different departments had diverse age groups and work activities, the staff members had different needs and interests.

With the cultural values of comradeship, employee-orientation, motivational leadership and commitment, the Armed Forces address the social dimension of meaningful coherence and purpose as outlined in the Sundsvall statement (Sundsvall Conference, 1991). The presented interviews with department heads indicate that WHP/OHM was seen as an additional social process that affects health.

However, the nonhierarchical WHP principles of participation in decision-making, decentralization of responsibilities, and communication/interconnection challenge the complex strictly hierarchical military structure. All interviewees talked about conflicts with the hierarchical structures and standardized processes that emerged with WHP implementation. Coordinators had to understand and honor the respective departmental structures. The recruitment of coordinators from the military culture was a beneficial choice. Adaptions according to deep cultural factors (e.g. values), and participation of community members have been suggested in culturally different contexts (Suárez-Reyes and Van den Broucke, 2016).

The coordinators used their functional, methodical, and especially social skills to establish a WHP network and integrate diverse actors on different hierarchical levels. Similarly, a long-term study on tasks and effectivity of safety experts in Germany identified ‘cooperative goal-orientation’ as the core facilitator, suggesting that safety experts are more successful if they can contact management directly and have a global understanding of employees’ health rather than a risk and compliance orientation (Trimpop *et al.*, 2012).

To integrate OSH into a comprehensive OHM, normative OSH practices should be designed to be more participative and to give employees room for negotiating terms if possible. Technically oriented OSH experts should be enabled to improve health and wellbeing (e.g. development of methodical and social skills). National regulations mandate the integration of OSH experts and risk assessment. Further, employers are legally responsible for assessing and managing (psychosocial) risks in the workplace. Thus, cross-linking of psychosocial risk assessment with WHP can close gaps between WHP and organization and its leadership. In this context, mandates and control functions of the workplace health committee must be clearly defined as a prerequisite to fulfill their transformational task.

The interviews provided convincing arguments that WHP can cause a mismatch between work demands and human resources for the supervisors. Many supervisors experience WHP/OHM not as advantageous for enhancing their own or subordinates’ health, but as lost time, productivity and flexibility. Employees have fewer hours per week available because they attend WHP or serve as WHP trainers. Until time discrepancies are resolved, middle managers will remain a barrier. Kliche and Touil (2011) did not directly address middle managers but subsume this issue under different transfer factors. Wierenga *et al.* (2013) and Rojatz *et al.* (2016) listed management support. Wierenga *et al.* (2013) particularly identified ‘lack of perceived management support by implementers on site’ as a barrier and ‘management willingness to provide release time from their usual duties to attend intervention’ as a facilitator.

Under organizational reorganizations, divisions in which managers and employees participate in WHP may lead to assumptions of an underutilized workforce. OHM investments require additional staffing as well as organizational and material resources. Further, assessment of readiness for change as an essential precursor to the successful implementation of WHP/OHM might be helpful (Zhang *et al.*, 2015).

Conclusions for the implementation of a sustainable OHM policy in a complex hierarchical organization are presented in Table 1. Following the comprehensive evaluation of the pilot study, the Federal Armed Forces took the decision to commit permanent resources to enable the development of a long-term comprehensive OHM.

**Table 1:** Conclusions for the implementation of an OHM in a complex hierarchical organization based on the content analysis of interviews with coordinators and their department heads who implemented a WHP pilot phase

• Implementing coordinators should be enabled to be authentic credible ambassadors for WHP/OHM.
• WHP/OHM can be successful only when the activities meet the varied needs and interests of different employees and their management. Thus, local department levels must be empowered to negotiate and develop cooperative structures.
• A conceptual and practical collaboration between WHP and OSH, and organization and leadership is necessary for the further development of a comprehensive OHM. OSH experts should be functionally integrated into the workplace health committee. Psychosocial risk assessment can close gaps between WHP and organization and its leadership.
• Middle management level supervisors may be better integrated if they receive open communication about compensation for the release time of their subordinates to attend WHP during working hours.
• Professional development should enable employees working in sport facilities or health and psychosocial services in complex organizations to receive credit for their career when they work to implement WHP/OHM (e.g. as coordinators).
• Managers and members of the workplace health committee should be offered further education to increase understanding, knowledge and commitment to WHP/OHM.
• For a healthy workplace, short WHP units should be an integral part of everyday working life, vocational training, and education.
• WHP marketing must enable flexible and provide solutions for very different conditions. For example, marketing might devise a logo or use internet/intranet portals for disseminating advertising and information.
• Environmental resources must be allocated to enable all stakeholders, whether employees or members of the highest OHM steering committee, to have access to an intranet IT-portal, possibly with staggered access rights. The portal should provide (i) the latest information, general background, and description of activities, (ii) a communication platform for exchanging ideas and good practices, and (iii) a stock exchange for sports and other materials. Linkage to cellular or smart phone would be beneficial.
• Interlinked process and outcome evaluations should accompany future large-scale OHM implementations.

OHM, occupational health management; OSH, occupational safety and health; WHP, workplace health promotion.

Future evaluations of complex WHP interventions need valid evaluation approaches that consider a step-wise approach and pre-implementation evaluation and assessment of the quality of implementation (Brug *et al.*, 2011). Regarding factors influencing WHP, the model of Rojatz *et al.* (Rojatz *et al.*, 2016) provides the most recent and comprehensive approach.
